# Numerical Analysis of an Unsteady, Electroviscous, Ternary Hybrid Nanofluid Flow with Chemical Reaction and Activation Energy across Parallel Plates

**DOI:** 10.3390/mi13060874

**Published:** 2022-05-31

**Authors:** Muhammad Bilal, A. El-Sayed Ahmed, Rami Ahmad El-Nabulsi, N. Ameer Ahammad, Khalid Abdulkhaliq M. Alharbi, Mohamed Abdelghany Elkotb, Waranont Anukool, Zedan A. S. A.

**Affiliations:** 1 Department of Mathematics, City University of Science and Information Technology, Peshawar 25000, KP, Pakistan; bilalchd345@gmail.com; 2 Mathematics Department, Faculty of Science, Taif University, P.O. Box 11099, Taif 21944, Saudi Arabia; ahsayed80@hotmail.com; 3 Center of Excellence in Quantum Technology, Faculty of Engineering, Chiang Mai University, Chiang Mai 50200, Thailand; waranont.a@cmu.ac.th; 4 Research Center for Quantum Technology, Faculty of Science, Chiang Mai University, Chiang Mai 50200, Thailand; 5 Department of Physics and Materials Science, Faculty of Science, Chiang Mai University, Chiang Mai 50200, Thailand; 6 Department of Mathematics, Faculty of Science, University of Tabuk, P.O. Box 741, Tabuk 71491, Saudi Arabia; anaudalur@ut.edu.sa; 7 Mechanical Engineering Department, College of Engineering, Umm Al-Qura University, Makkah 21955, Saudi Arabia; kamharbi@uqu.edu.sa; 8 Mechanical Engineering Department, College of Engineering, King Khalid University, Abha 61421, Saudi Arabia; melkotb@kku.edu.sa (M.A.E.); ahafedh@kku.edu.sa (Z.A.S.A.); 9 Mechanical Engineering Department, College of Engineering, Kafrelsheikh University, Kafrelsheikh 33516, Egypt; 10 Mechanical Engineering Department, Collage of Engineering at Shoubra, Benha University, Cairo 11629, Egypt

**Keywords:** ternary hybrid nanofluids, activation energy, Darcy–Forchheimer flow, electroviscous effect, electric potential, parametric continuation method

## Abstract

Despite the recycling challenges in ionic fluids, they have a significant advantage over traditional solvents. Ionic liquids make it easier to separate the end product and recycle old catalysts, particularly when the reaction media is a two-phase system. In the current analysis, the properties of transient, electroviscous, ternary hybrid nanofluid flow through squeezing parallel infinite plates is reported. The ternary hybrid nanofluid is synthesized by dissolving the titanium dioxide (TiO_2_), aluminum oxide (Al_2_O_3_), and silicon dioxide (SiO_2_) nanoparticles in the carrier fluid glycol/water. The purpose of the current study is to maximize the energy and mass transfer rate for industrial and engineering applications. The phenomena of fluid flow is studied, with the additional effects of the magnetic field, heat absorption/generation, chemical reaction, and activation energy. The ternary hybrid nanofluid flow is modeled in the form of a system of partial differential equations, which are subsequently simplified to a set of ordinary differential equations through resemblance substitution. The obtained nonlinear set of dimensionless ordinary differential equations is further solved, via the parametric continuation method. For validity purposes, the outcomes are statistically compared to an existing study. The results are physically illustrated through figures and tables. It is noticed that the mass transfer rate accelerates with the rising values of Lewis number, activation energy, and chemical reaction. The velocity and energy transfer rate boost the addition of ternary NPs to the base fluid.

## 1. Introduction

The squeezing flow is important in lubrication equipment, polymer processing, molding compaction, and injection, all of which use hydrodynamical technologies generated from moving surfaces. Jackson [1] highlights the connection between loaded bearings and compressing flow operation in engines, which includes the occurrence of adhesion. Muhammad et al. [2] describe the unsteady squeezed flow of a hybrid nanofluid (HNF), made up of CNTs and CuO, using a numerical technique. The fluid velocity improves as the squeezing intensity and volume fraction of nanomaterials increases. Ramesh et al. [3] explore the unsteady squeezing flow of hydromagnetic and Casson NFs using enclosed parallel disks. Selimefendigil et al. [4] conduct a numerical analysis of nanofluid forced circulation inside a branching channel, under the effect of a constant magnetic flux. Xu et al. [5] study stable power law NF flow, including gyrotactic microorganisms that transmit energy between two parallel plates. Shuaib et al. [6] use 3D flow characteristics to display a 3D NF flow across two contemporaneous circular plates. Their purpose is to see how magnetic induction affects NF flow with heat transmission qualities in the long run. To examine the MHD NF flow through extendable spinning discs, Ahmadian et al. [7] employ the numerical approach bvp4c. The disc stretching process, according to the findings, opposes the flow tendency. Bilal et al. [8] consider the effects of MHD and EHD parameters on the flow of water-based hybrid NFs across two circular plates. With the growing Reynolds number, magnetic, and electric effects, heat transmission is estimated to rise [9,10]. Khan et al. [11] use Fourier’s and Fick’s laws to explore the channel flow of a second-grade viscoelastic fluid between two plates, formed by a vibrating wall with mass and energy transport characteristics. Alsallami et al. [12] develop an Maxwell nanofluid flow with arrhenius activation energy over a rotating disk. Dawar et al. [13] deploy freshwater as a conventional fluid across two surfaces in order to study copper oxide and copper nanoparticles. The results demonstrate that the particulate concentration factor has a dual influence on velocity distribution. Some recent studies are found in [14,15,16,17].

A hybrid nanofluid is a new type of fluid that outperforms when compared to regular fluids, such as ethyl alcohol, water, nanofluids, and ethylene, during energy transitions. HNFs have a huge spectrum of thermal properties, including the ability to freeze at high temperatures [18,19,20]. Hybrid NFs are used in energy generation, heat transfers, heat pumps, air conditioners, the automotive industry, electrical appliances, turbines, nuclear reactors, broadcasting, spacecraft, and biotechnology [21]. Coolant and freezing solutions containing ethylene glycol and water provide significant advantages, such as reducing corrosion and acid deterioration, as well as suppressing the growth of most bacteria and fungi. In the industry, ethylene glycol and water mixes are referred to as glycol concentrates, mixtures, solutions, and compounds. We employed the TiO_2_, SiO_2_, and Al_2_O_3_ NPs in the working fluid in this study. TiO_2_ is a white inorganic material that has been used for over a century in a variety of foodstuffs [22]. It is the brightest and whitest pigment known, with reflective qualities, and the ability to absorb and scatter UV radiation [23,24]. Silica is one of the most abundant class of compounds, and because of its inexpensive cost of manufacture, high surface area, and wettability, it has the potential to be an outstanding choice for commercial usage [25,26]. Zhang et al. [27] inspect the entropy maximization in a hydromagnetic HNF flow of SiO2 and (MoS2 (molybdenum disulfide) NPs flowing toward a stretchy surface. Ahmed et al. [28] inspect the Ag–MgO HNF flow with heat propagation generated by a curved spinning disc that rotates in three dimensions, both vertically and horizontally. Chu et al. [29] evaluate flow kinematics and heat transfer from the perspective of TiO_2_ and Al_2_O_3_ NPs used to rise the thermal characteristics of the base fluid. The efficiency of an HNF containing TiO_2_ and MWCNTs is analyzed by Chu et al. [30]. Long et al. [31] assess the covalent bonding reactivity of a hybrid ferrofluid flow containing Fe_3_O_4_ and CoFe_2_O_4_ NPs, in both crosswise and streamwise positions. They made CoFe_2_O_4_ NPs with a well-defined mesoporous dominant structure using hybrid CoFeHCF (hexacyanoferrate) NPs as a substrate. Their research shows a new way to make CoFe_2_O_4_ nano catalysts for pollutant degradation and promotes the usage of CoFeHCF in the ecosystem. Ullah et al. [32] develop a theoretical formulation for a nonlinear magnetic TiO_2_ NF flow through an expanding cylinder Darcy-permeable medium. Shah et al. [33] provide a concise overview of most of TiO_2′_s notable properties, as well as a summary of innovative thermal applications, including its high refractive index, overwhelmingly high boiling and melting points, high stiffness and hardness, ability to absorb or reflect ultraviolet radiation, and photocatalytic nature. Recently, many researchers reported significant contributions to ternary hybrid nanofluid flow [34,35,36,37].

Magnetism is one of the key significant features of engineering and drug distribution due to its wide range of applications; heat exchangers, clutches, and compressors, to name a few major commodities, are all modified by the combination of flowing fluid under a magnetic field [38,39,40]. Magnetic fields have the potential to regulate and make the working temperature of a number of industrial devices more convenient. Magnetic fields are used in interplanetary and extremely high magneto applications, as well as in aerodynamics and chemical chemistry. The strength and scattering of the applied magnetics have an impact on the flow behavior. To describe the flow properties under the upshot of magnetic flux, many researchers committed to fluid mechanics. Hayat et al. [41] look at how specific heat and a produced magnetosphere affected the sinusoidal flow of an HNF flow, via a lateral tube. Raza et al. [42] examine the effect of a molybdenum disulfide nanofluid exchanger and MHD on free convective flow through a channel. Dezfulizadeh et al. [43] explore the performance of MHD ternary HNF flow in a thermal exchanger, using a unique compound-perverted turbulator and spiral rotors. Per the PEC indices, the twisted spinning bar with ellipsoidal surface obstacles has the maximum exergy effectiveness, increasing by 7% in Re, to 12,000. References [44,45,46] contains some recent literature on MHD HNF.

The present study aimed to numerically examine the cumulative influence of the electromagnetic force, chemical reaction, suction/injection, inertia force, activation energy, ionized fluid, and magnetic field on the squeezing flow of ternary hybrid nanofluids across parallel plates. We supposed that the lower plate is permeable and stretching with a uniform velocity. For this purpose, the phenomena were modeled and formulated in the form of a system of PDEs, which are solved through the parametric continuation method. The results are shown through figures and tables. In the above-described, ionized, ternary nanofluid model, the effects of activation energy, heat source, and chemical reactions in the uses of ternary hybrid nanofluid are the main novelty of the proposed model. Furthermore, in the next section, the problem was articulated, resolved, and discoursed.

## 2. Governing Equations

**Figure 1:**
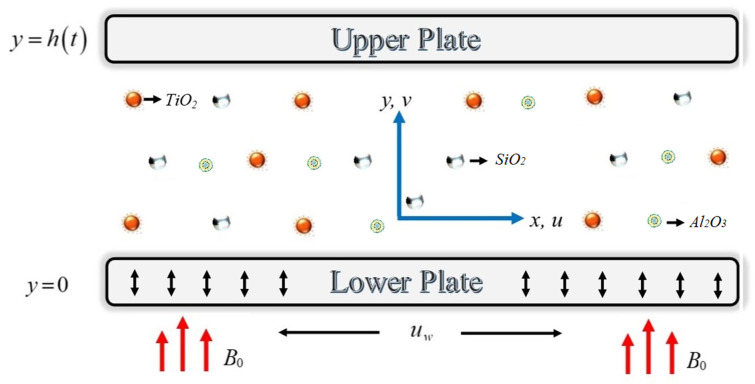
Electroviscous fluid flow across two parallel plates.

The ternary hybrid nanofluid flow across two parallel infinite plates, consisting of titanium dioxide, silicon dioxide, and aluminum oxide is reported. The flow mechanism is graphically depicted in Figure 1. The upper plate is located at $y=h(t)=\sqrt{\frac{\left(1-\alpha t\right)\nu_{bf}}{b}}$, which fluctuates downwards with the velocity $\frac{dh}{dt}=\frac{-\alpha }{2}\sqrt{\frac{\nu_{bf}}{b\left(1-\alpha t\right)}}$. The lower plate is permeable, which allows suction/injection effect, signified as $V_{w}=\frac{-V_{0}}{\left(1-\alpha t\right)}.$ Both plates are assumed at constant temperatures *T_1_* and *T_2_*. The lower plate is expanding with the linear velocity $u_{w}=\frac{-bx}{\left(1-\alpha t\right)}.$ Furthermore, the time-dependent magnetic field is characterized as $H=\frac{B_{0}}{\left(1-\alpha t\right)}$. The basic flow equations are communicated as [47,48,49]:

(1) $$\frac{\partial u}{\partial x}+\frac{\partial v}{\partial y}=0,$$

(2) $$\frac{\partial U}{\partial t}+u\frac{\partial U}{\partial x}+v\frac{\partial U}{\partial y}=\frac{\mu_{hnf}}{\rho_{hnf}}\frac{\partial^{2}U}{\partial y^{2}}-\frac{\sigma_{hnf}}{\rho_{hnf}}H^{2}U-\frac{\mu_{hnf}}{\rho_{hnf}}\frac{U}{K^{*}}\;-FrU^{2}-\left(n^{+}-n^{-}\right)\frac{BK^{2}\mu_{hnf}}{\rho_{hnf}}\frac{\partial W}{\partial x},$$

(3) $$\frac{\partial^{2}W}{\partial x^{2}}+\frac{\partial^{2}W}{\partial y^{2}}=\frac{K}{2}\left(n^{+}-n^{-}\right),$$

(4) $$\frac{\partial n^{+}}{\partial t}+u\frac{\partial n^{+}}{\partial x}+v\frac{\partial n^{+}}{\partial y}=\frac{\mu_{hnf}}{\rho_{hnf}Sc}\left(\frac{\partial^{2}n^{+}}{\partial y^{2}}+\frac{\partial W}{\partial x}\frac{\partial n^{+}}{\partial x}+\frac{\partial W}{\partial y}\frac{\partial n^{+}}{\partial y}\;+n^{+}\frac{\partial^{2}W}{\partial y^{2}}\right),$$

(5) $$\frac{\partial n^{-}}{\partial t}+u\frac{\partial n^{-}}{\partial x}+v\frac{\partial n^{-}}{\partial y}=\frac{\mu_{hnf}}{\rho_{hnf}Sc}\left(\frac{\partial^{2}n^{-}}{\partial y^{2}}+\frac{\partial W}{\partial x}\frac{\partial n^{-}}{\partial x}+\frac{\partial W}{\partial y}\frac{\partial n^{-}}{\partial y}\;+n^{-}\frac{\partial^{2}W}{\partial y^{2}}\right),$$

(6) $$\frac{\partial T}{\partial t}+u\frac{\partial T}{\partial x}+v\frac{\partial T}{\partial y}=\frac{K_{hnf}}{{\left(\rho C_{p}\right)}_{hnf}}\frac{\partial^{2}T}{\partial y^{2}}+\frac{Q_{0}}{{\left(\rho C_{p}\right)}_{hnf}}\left(T-T_{0}\right),$$

(7) $$\frac{\partial C}{\partial t}+u\frac{\partial C}{\partial x}+v\frac{\partial C}{\partial y}=D_{B}\frac{\partial^{2}C}{\partial y^{2}}+\frac{D_{T}}{T_{1}}\frac{\partial^{2}T}{\partial y^{2}}-k_{r}^{2}\left(C-C_{0}\right){\left(\frac{T}{T_{\infty }}\right)}^{n}\exp\left(-\frac{E_{a}}{\kappa T}\right).$$

Equation (1) is the continuity equation, Equation (2) is the momentum equation with electroviscous and uniform magnetic effect, Equation (3) is the Poisson equation, Equations (4) and (5) are Nernst–Planck equations, while Equations (6) and (7) are the energy and mass distribution equations, respectively.

In Equations (1)–(7), *n*^−^ and *n*^+^ are the negative and positive charged ions; $U=\frac{\partial v}{\partial x}-\frac{\partial u}{\partial y}$ are the associate’s condition for the upper and lower plate; *T* and *C* are the temperature and the concentration, respectively; $Fr=\frac{C_{b}^{*}}{\sqrt{K^{*}}}$ is the porous media non-inertial coefficient, where K* and $C_{b}^{*}$ are the permeability factor and drag force constant, respectively; *Q*_0_ is the heat generation term; $K^{2}=\frac{2z^{2}e^{2}n_{0}}{\varepsilon_{0}\varepsilon k_{b}T}$ is the inverse Debye factor; *W* is the electric potential of ions; and $B=\frac{\rho k^{2}T^{2}\varepsilon_{0}\varepsilon }{2z^{2}e^{2}\mu^{2}}$ is fixed at a constant temperature. $E_{a}$ is the activation energy, and *k_T_* is the chemical reaction rate. Furthermore, ${\left(\rho C_{p}\right)}_{Thnf},\;\;\;\mu_{Thnf},\;\;\;\sigma_{Thnf},\;\;\kappa_{Thnf}\;\;\;\mathrm{and}\;\;\;\;\rho_{Thnf}$ are the heat capacity, dynamic viscosity, electrical conductivity, thermal conductivity, and density of ternary HNF, respectively.

The boundary conditions are:

(8) $$\begin{array}{l} u=\lambda_{1}\frac{bx}{\left(1-\alpha t\right)},\;\;\;v=-\frac{V_{0}}{\left(1-\alpha t\right)},\;\;\;T=T_{1},\;\;\;C=C_{1},\;\;\;W=0,\;\;\;n^{-}=n^{+}=0\;\;\;\;\;\mathrm{at}\;\;\;\;y=0\\ u=0,\;\;\;v=-\frac{dh\left(t\right)}{dt},\;\;\;T=T_{2},\;\;\;C=C_{2},\;\;\;W=\frac{x}{l^{2}\left(1-\alpha t\right)},\;\;\;n^{-}=n^{+}=\frac{\alpha }{\nu_{b_{f}}\left(1-\alpha t\right)}\;\;\;\;\;\mathrm{at}\;\;\;\;y=h\left(t\right). \end{array}$$

The similarity variables are:

(9) $$\begin{array}{l} \Psi =\sqrt{\frac{b\upsilon_{bf}}{\left(1-\alpha t\right)}}x\;f\left(\eta \right),\;\;u=\frac{bx}{\left(1-\alpha t\right)}f^{\prime }\left(\eta \right),\;\;v\;=-\sqrt{\frac{b\upsilon_{bf}}{\left(1-\alpha t\right)}}f\left(\eta \right),\;\;\theta \left(\eta \right)=\frac{T-T_{1}}{T_{2}-T_{1}},\;\;\phi \left(\eta \right)=\frac{C-C_{1}}{C_{2}-C_{1}},\\ W=\frac{x}{l^{2}\left(1-\alpha t\right)}P\left(\eta \right),\;\;\;\;n^{-}=\frac{\alpha }{\upsilon_{bf}\left(1-\alpha t\right)}H\left(\eta \right),\;\;\;n^{+}=\frac{\alpha }{\upsilon_{bf}\left(1-\alpha t\right)}G\left(\eta \right),\;\;\;\;\eta =y\sqrt{\frac{b}{\upsilon_{bf}\left(1-\alpha t\right)}}.\;\; \end{array}$$

Therefore, the transformed set of ODEs is:

(10) $$f^{iv}=\frac{\vartheta_{1}}{\vartheta_{2}}\left(\frac{Sq}{2}\left(\eta f^{\prime \prime \prime }+3f^{\prime }\right)-ff^{\prime \prime \prime }+f^{\prime }f^{\prime \prime }-Fr{f^{\prime \prime }}^{2}\right)-\frac{\vartheta_{3}}{\vartheta_{2}}Mf^{\prime \prime }+K_{1}^{*}f^{\prime \prime }+BK^{2}RH\left(G-H\right),$$

(11) $$p^{\prime \prime }=-\frac{1}{2}K^{2}\delta_{1}\left(G-H\right),$$

(12) $$g^{\prime \prime }=\frac{\;\vartheta_{1}}{\vartheta_{2}}\left(\frac{Sq}{2}\left(\eta g^{\prime }+2g\right)-fg^{\prime }\right)Sc-\frac{1}{\delta_{1}}\left(g^{\prime }p^{\prime }-\frac{K^{2}\delta_{1}}{2}\left(g^{2}-gh\right)\right),$$

(13) $$h^{\prime \prime }=\frac{\;\vartheta_{1}}{\vartheta_{2}}\left(\frac{Sq}{2}\left(\eta h^{\prime }+2h\right)-fh^{\prime }\right)Sc-\frac{1}{\delta_{1}}\left(h^{\prime }p^{\prime }-\frac{K^{2}\delta_{1}}{2}\left(gh-h^{2}\right)\right),$$

(14) $$\theta^{\prime \prime }=\frac{\;\vartheta_{4}}{\vartheta_{35}}\left(\frac{Sq\;Pr}{2}\eta \theta^{\prime }-Prf\theta^{\prime }-\frac{Pr}{\vartheta_{4}}Q\theta \right),$$

(15) $$\phi^{\prime \prime }=\frac{Sq\;Le}{2}\eta \phi^{\prime }-Lef\phi^{\prime }-\frac{Nt}{Nb}\frac{\;\vartheta_{4}}{\vartheta_{5}}\left(\frac{Sq\;Pr}{2}\eta \theta^{\prime }-Prf\theta^{\prime }-\frac{Pr}{\vartheta_{4}}Q\theta \right)-Sc\sigma {\left(1+\delta \theta \right)}^{n}\phi \;\;\exp\left(-\frac{E}{1+\delta \theta }\right).$$

Here, $\vartheta_{1}=\frac{\rho_{Thnf}}{\rho_{bf}},\;\;\;\;\vartheta_{2}=\frac{\mu_{Thnf}}{\mu_{bf}},\;\;\;\;\vartheta_{3}=\frac{\sigma_{Thnf}}{\sigma_{bf}},\;\;\;\;\vartheta_{4}=\frac{{\left(\rho Cp\right)}_{Thnf}}{{\left(\rho Cp\right)}_{bf}},\;\;\;\;\vartheta_{5}=\frac{\kappa_{Thnf}}{\kappa_{bf}}.$ *M* is the magnetic term, *Pr* is the Prandtl number, *Sq* is the squeezing constraint, $K_{1}^{*}$ is the local porosity term, λ > 0 is the stretching parameter for the lower plate (λ = 0) for the fixed plate), *Fr* is the Forchheimer number, *Sc* is the Schmidt number, *Nt* is the thermophoresis constant, *S* is the suction/injection term, *Le* is the Lewis number, *Q* is the heat source/sink term, *Nb* is the Brownian motion constant, *E* is the Arrhenius activation energy coefficient, and σ is the chemical reaction term, defined as:

(16) $$\begin{array}{l} M=\frac{\sigma_{bf}B_{0}^{2}}{b\rho_{bf}},\;\;\;Pr=\frac{\mu_{bf}Cp_{bf}}{\kappa_{bf}},\;\;Sq=\frac{\alpha }{b},\;\;K_{1}^{*}=\frac{\upsilon_{bf}\left(1-\alpha t\right)}{K^{*}b},\;\;\;Sc=\frac{\mu_{bf}}{\rho_{bf}D},\;\;Nt=\frac{D_{T}\left(T_{2}-T_{1}\right)}{T_{1}\upsilon_{bf}},\\ S=\frac{V_{0}}{lb},\;\;Le=\frac{\upsilon_{bf}}{D_{B}},\;\;\delta_{1}=\frac{\alpha^{2}}{l^{2}},\;\;Q=\frac{Q_{0}}{b{\left(\rho C_{p}\right)}_{bf}},\;\;Nb=\frac{D_{B}\left(C_{2}-C_{1}\right)}{\upsilon_{bf}},\;\;\;E=\frac{E_{a}}{\kappa T_{\infty }},\;\;\;\;\sigma =\frac{k_{T}^{2}}{c}. \end{array}$$

The transform boundary conditions are:

(17) $$\begin{array}{l} f^{\prime }\left(0\right)=\lambda ,\;\;f\left(0\right)=S,\;\;\theta \left(0\right)=\delta ,\;\;\phi \left(0\right)=\omega ,\;\;p\left(0\right)=0,\;\;g\left(0\right)=0,\;\;h\left(0\right)=0\;\;\;\;at\;\;\;y=0\\ f^{\prime }\left(1\right)=0,\;\;f\left(1\right)=\frac{Sq}{2},\;\;\;\theta \left(1\right)=1,\;\;\;\phi \left(1\right)=1,\;\;\;p\left(1\right)=1,\;\;\;h\left(1\right)=1,\;\;\;g\left(1\right)=1\;\;\;\;at\;\;\;y=1. \end{array}\}$$

The Nusselt number and the skin friction are characterized as:

(18) $$\begin{array}{l} Re_{x}^{1/2}C_{f_{u}}=\frac{\mu_{Thnf}}{\mu_{bf}}f^{\prime \prime }\left(1\right),\;\;\;\;\;Re_{x}^{1/2}C_{f_{l}}=\frac{\mu_{Thnf}}{\mu_{bf}}f^{\prime \prime }\left(0\right),\\ Re_{x}^{-1/2}N_{u_{u}}=-\frac{\kappa_{Thnf}}{\kappa_{bf}}\theta^{\prime }\left(1\right),\;\;\;\;\;Re_{x}^{-1/2}N_{u_{l}}=-\frac{\kappa_{Thnf}}{\kappa_{bf}}\theta^{\prime }\left(0\right). \end{array}\}$$

where $Re_{x}=\frac{xU_{w}}{\upsilon_{bf}}.$

## 3. Numerical Solution

This section shows how to use the algorithm of the numerical scheme to solve the numerical solutions of the suggested mathematical model. The main steps for dealing with the parametric continuation method scheme and future direction are as follows [50,51,52,53,54,55,56,57]:

Step 1: Simplifying the modeled equations to 1st order:

(19) $$\begin{array}{l} \hbar_{1}=f,\;\;\;\hbar_{2}=f^{\prime },\;\;\;\hbar_{3}=f^{\prime \prime },\;\;\;\hbar_{4}=f^{\prime \prime \prime },\;\;\;\hbar_{5}=p,\;\;\;\hbar_{6}=p^{\prime },\;\;\;\hbar_{7}=g,\\ \hbar_{8}=g^{\prime },\;\;\;\hbar_{9}=h,\;\;\;\hbar_{10}=h^{\prime },\;\;\;\hbar_{11}=\theta ,\;\;\;\hbar_{12}=\theta^{\prime },\;\;\;\hbar_{13}=\phi ,\;\;\;\hbar_{14}=\phi^{\prime }.\; \end{array}\}$$

By putting Equation (19) in Equations (10)–(15) and (17), we achieve:

(20) $${\hbar^{\prime }}_{4}=\frac{\vartheta_{1}}{\vartheta_{2}}\left(\frac{Sq}{2}\left(\eta \hbar_{4}+3\hbar_{2}\right)-\hbar_{1}\hbar_{4}+\hbar_{2}\hbar_{3}-Fr\hbar_{3}^{2}\right)-\frac{\vartheta_{3}}{\vartheta_{2}}Mf^{\prime \prime }+K_{1}^{*}\hbar_{3}+BK^{2}R\hbar_{9}\left(\hbar_{7}-\hbar_{9}\right),$$

(21) $${\hbar^{\prime }}_{6}=-\frac{1}{2}K^{2}\delta_{1}\left(\hbar_{7}-\hbar_{9}\right),$$

(22) $${\hbar^{\prime }}_{8}=\frac{\;\vartheta_{1}}{\vartheta_{2}}\left(\frac{Sq}{2}\left(\eta \hbar_{8}+2\hbar_{7}\right)-\hbar_{1}\hbar_{8}\right)Sc-\frac{1}{\delta_{1}}\left(\hbar_{8}\hbar_{6}-\frac{K^{2}\delta_{1}}{2}\left(\hbar_{7}{}^{2}-\hbar_{7}\hbar_{9}\right)\right),$$

(23) $${\hbar^{\prime }}_{10}=\frac{\;\vartheta_{1}}{\vartheta_{2}}\left(\frac{Sq}{2}\left(\eta \hbar_{10}+2\hbar_{9}\right)-\hbar_{1}\hbar_{10}\right)Sc-\frac{1}{\delta_{1}}\left(\hbar_{10}\hbar_{6}-\frac{K^{2}\delta_{1}}{2}\left(\hbar_{7}\hbar_{9}-\hbar_{9}{}^{2}\right)\right),$$

(24) $${\hbar^{\prime }}_{12}=\frac{\;\vartheta_{4}}{\vartheta_{35}}\left(\frac{Sq\;Pr}{2}\eta \hbar_{12}-Pr\hbar_{1}\hbar_{12}-\frac{Pr}{\vartheta_{4}}Q\hbar_{11}\right),$$

(25) $${\hbar^{\prime }}_{14}=\frac{Sq\;Le}{2}\eta \hbar_{14}-Le\hbar_{1}\hbar_{14}-\frac{Nt}{Nb}\frac{\;\vartheta_{4}}{\vartheta_{5}}\left(\begin{array}{l} \frac{Sq\;Pr}{2}\eta \hbar_{12}-Pr\\ \hbar_{1}\hbar_{12}-\frac{Pr}{\vartheta_{4}}Q\hbar_{11} \end{array}\right)-Sc\sigma {\left(1+\delta \theta \right)}^{n}\hbar_{13}\;\;\exp\left(-\frac{E}{1+\delta \hbar_{11}}\right).$$

with the corresponding boundary conditions:

(26) $$\begin{array}{l} \hbar_{2}\left(0\right)=\lambda ,\;\;\hbar_{1}\left(0\right)=S,\;\;\;\hbar_{11}\left(0\right)=\delta ,\;\;\hbar_{13}\left(0\right)=\omega ,\;\;\hbar_{5}\left(0\right)=0,\;\;\hbar_{7}\left(0\right)=0,\;\;\hbar_{9}\left(0\right)=0\;\;\;\;at\;\;\;y=0\\ \hbar_{2}\left(1\right)=0,\;\;\hbar_{1}\left(1\right)=\frac{Sq}{2},\;\;\;\hbar_{11}\left(1\right)=1,\;\;\;\hbar_{13}\left(1\right)=1,\;\;\;\hbar_{5}\left(1\right)=1,\;\;\;\hbar_{7}\left(1\right)=1,\;\;\;\hbar_{9}\left(1\right)=1\;\;\;\;at\;\;\;y=1. \end{array}\}$$

Step 2: Introducing the embedding parameter *p*:

(27) $${\hbar^{\prime }}_{4}=\frac{\vartheta_{1}}{\vartheta_{2}}\left(\frac{Sq}{2}\left(\eta \hbar_{4}+3\hbar_{2}\right)-\hbar_{1}\left(\hbar_{4}-1\right)p+\hbar_{2}\hbar_{3}-Fr\hbar_{3}^{2}\right)-\frac{\vartheta_{3}}{\vartheta_{2}}Mf^{\prime \prime }+K_{1}^{*}\hbar_{3}+BK^{2}R\hbar_{9}\left(\hbar_{7}-\hbar_{9}\right),$$

(28) $${\hbar^{\prime }}_{6}=-\frac{1}{2}K^{2}\delta_{1}\left(\hbar_{7}-\hbar_{9}\right),$$

(29) $${\hbar^{\prime }}_{8}=\frac{\;\vartheta_{1}}{\vartheta_{2}}\left(\frac{Sq}{2}\left(\eta \hbar_{8}+2\hbar_{7}\right)-\hbar_{1}\left(\hbar_{8}-1\right)p\right)Sc-\frac{1}{\delta_{1}}\left(\hbar_{8}\hbar_{6}-\frac{K^{2}\delta_{1}}{2}\left(\hbar_{7}{}^{2}-\hbar_{7}\hbar_{9}\right)\right),$$

(30) $${\hbar^{\prime }}_{10}=\frac{\;\vartheta_{1}}{\vartheta_{2}}\left(\frac{Sq}{2}\left(\eta \hbar_{10}+2\hbar_{9}\right)-\hbar_{1}\left(\hbar_{10}-1\right)p\right)Sc-\frac{1}{\delta_{1}}\left(\hbar_{10}\hbar_{6}-\frac{K^{2}\delta_{1}}{2}\left(\hbar_{7}\hbar_{9}-\hbar_{9}{}^{2}\right)\right),$$

(31) $${\hbar^{\prime }}_{12}=\frac{\;\vartheta_{4}}{\vartheta_{35}}\left(\frac{Sq\;Pr}{2}\eta \hbar_{12}-Pr\hbar_{1}\left(\hbar_{12}-1\right)p-\frac{Pr}{\vartheta_{4}}Q\hbar_{11}\right),$$

(32) $${\hbar^{\prime }}_{14}=\frac{Sq\;Le}{2}\eta \left(\hbar_{14}-1\right)p-Le\hbar_{1}\hbar_{14}-\frac{Nt}{Nb}\frac{\;\vartheta_{4}}{\vartheta_{5}}\left(\begin{array}{l} \frac{Sq\;Pr}{2}\eta \hbar_{12}-Pr\\ \hbar_{1}\hbar_{12}-\frac{Pr}{\vartheta_{4}}Q\hbar_{11} \end{array}\right)-Sc\sigma {\left(1+\delta \theta \right)}^{n}\hbar_{13}\;\;\exp\left(-\frac{E}{1+\delta \hbar_{11}}\right).$$

Step 3: Differentiating by parameter ‘*p*’:

(33) V′=ΔV+R,

where Δ is the coefficient matrix.

(34) $$V=\frac{d\hbar_{i}}{d\tau }$$

where *i* = 1, 2,…, 11.

Step 4: Apply the Cauchy principal:

(35) V=aU+W,

where W and U are the indefinite vector functions.

(36) $$U^{\prime }=aU,$$

(37) W′=ΔW+R,

By putting the approximate solution Equation (26) into the original Equation (24), we obtain:

(38) (aU+W)′=Δ(aU+W)+R,

Step 5: Solving the Cauchy problems:

(39) Ui+1−UiΔη=ΔUi+1,    Wi+1−WiΔη=ΔWi+1.

Finally, we obtain:

(40) Ui+1=(I−ΔΔη)−1Ui,        Wi+1=(I−ΔΔη)−1(Wi+ΔηR).

## 4. Results and Discussion

This section reveals the physical description of the obtained results in form of figures and tables for velocity, energy, and mass transfer profiles versus several physical constraints. The default parametric values used for the simulation of modeled equations are: *ϕ*_1_ = *ϕ*_2_ = *ϕ*_3_ = 0.01, *S* = 1.0, *Fr* = 0.5, *S* = 1.0 & −1.0, *Sc* = 0.1, *Le* = 0.4, *Sq* = 0.5, *E* = 1.0, *M* = 0.3, *Nt* = *Nb* = 0.1, *σ* = 0.5, and *Q* = 0.2. The following observations are noticed.

**Figure 2:**
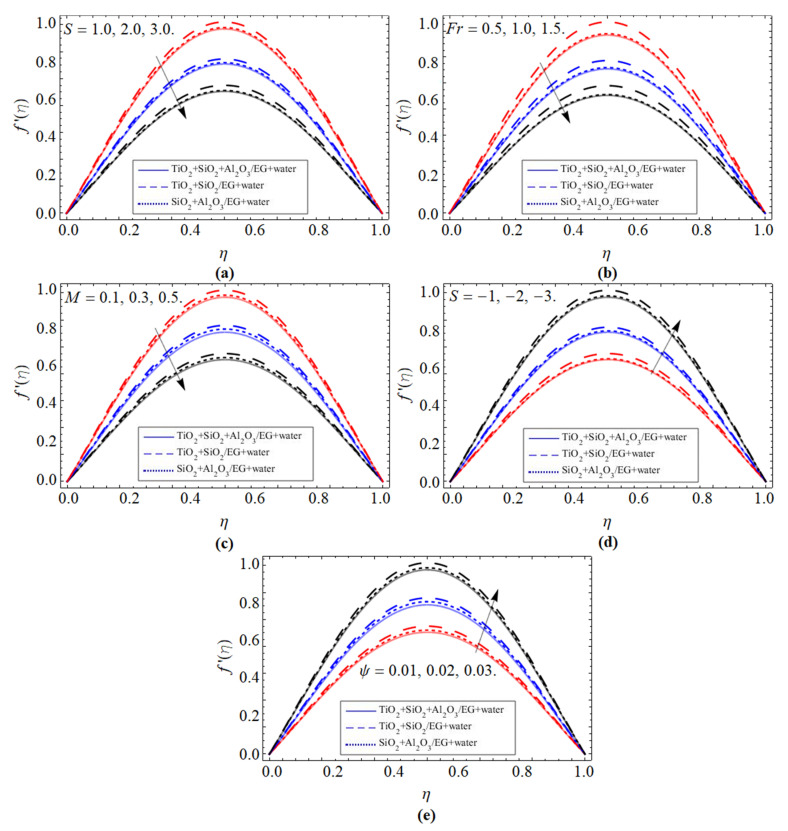
The tendency of velocity profile (*f*′(*η*)) versus (**a**) suction term *S > 0*, (**b**) Darcy–Forchheimer *Fr*, (**c**) magnetic field *M*, (**d**) injection *S < 0*, and (**e**) volume friction of ternary nanoparticles $\Psi =\left(\phi_{1}=\phi_{2}=\phi_{3}\right)$.

Velocity Profile (*f*′(*η*)):

Figure 2a–e displays the tendency of velocity profile(*f*′(*η*)) versus suction parameter *S >* 0, Darcy–Forchheimer *Fr*, magnetic field *M*, injection *S* < 0, and volume friction of ternary nanoparticles $\Psi =\left(\phi_{1}=\phi_{2}=\phi_{3}\right)$, respectively. Figure 2a–c shows that the velocity field is lessened with the upshot of suction parameter, Darcy–Forchheimer, and magnetic field. Physically, the rising effect of the suction factor diminishes the motion of fluid particles, which causes a reduction in the velocity profile, as shown in Figure 2a. The permeability of the plate surface enhances with the variation of the Darcy effect, which also encourages more suction from the plate surface and, as a result, fluid velocity (*f*′(*η*)) declines, as depicted in Figure 2b. The resistive force, which is created due to the magnetic effect *M*, opposes the fluid motion, similarly also deducing the velocity boundary layer, as shown in Figure 2c.

Figure 2d,e illustrate that the velocity distribution accelerates with the flourishing values of injection and volume friction of ternary nanoparticles. Physically, due to the injection effect of fluid particles, the fluid moves fast; as a consequence, the velocity of fluid flow elevates, as elaborated in Figure 2d. The addition of ternary nanoparticles (TiO_2_, SiO_2_, Al_2_O_3_) to the base fluid magnifies its thermal conduction, which also causes the inclination in the velocity field, as revealed in Figure 2e.

**Figure 3:**
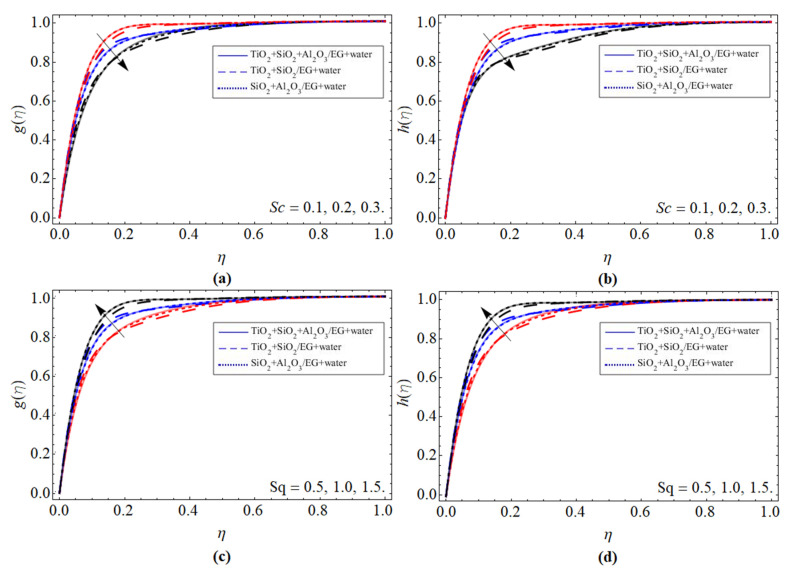
The tendency of electric field (*g*(*η*), *h*(*η*)) versus (**a**,**b**) Schmidt number *Sc*, (**c**,**d**) squeezing term *Sq*.

Electric Field (*g*(*η*), *h*(*η*)):

Figure 3a–d report the presentation of the electric field (*g*(*η*), *h*(*η*)) profile versus the Schmidt number *Sc*, and squeezing term *Sq*, respectively. The kinetic viscosity of fluid enhances with the effect of the Schmidt number, which diminishes the molecular dissemination, and causes the lessening of the electric field (*g*(*η*), *h*(*η*)), as shown in Figure 3a,b. The influence of the squeezing variable fluctuates the fluid particles, which enhances its velocity, and as a result, the electric profile is also boosted, as elaborated in Figure 3c,d.

**Figure 4:**
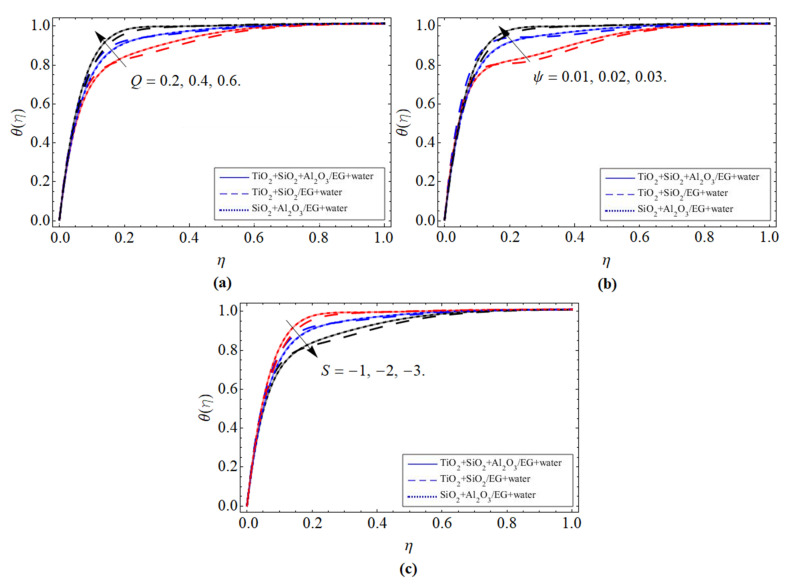
The tendency of energy profile *θ*(*η*) versus (**a**) heat source *Q*, (**b**) volume friction of ternary nanoparticles Ψ, and (**c**) injection *S* < 0.

Energy Profile *θ*(*η*):

Figure 4a–c represents the tendency of energy profile *θ*(*η*) versus the heat source *Q*, volume friction of ternary nanoparticles Ψ, and injection *S* < 0 constraints, respectively. As a consequence of the heat generation term, thermal energy is generated inside the fluid flow, which causes the elevation of the energy profile *θ*(*η*), as seen in Figure 4a. Figure 4b illustrates that the inclusion of nanomaterials (TiO_2_, SiO_2_, Al_2_O_3_) in the base fluid augments the thermal conduction of the base fluid, as well as reduces the average heat capacity, because the specific heat capacity of ethylene glycol/water is much higher than ternary nanoparticles. This is why the energy propagation rate of ternary nanofluid magnifies with the rising quantity in the concentration of nanoparticles Ψ. The energy transfer rate of ternary NFs declines with the upshot of the injection term, as shown in Figure 4c.

**Figure 5:**
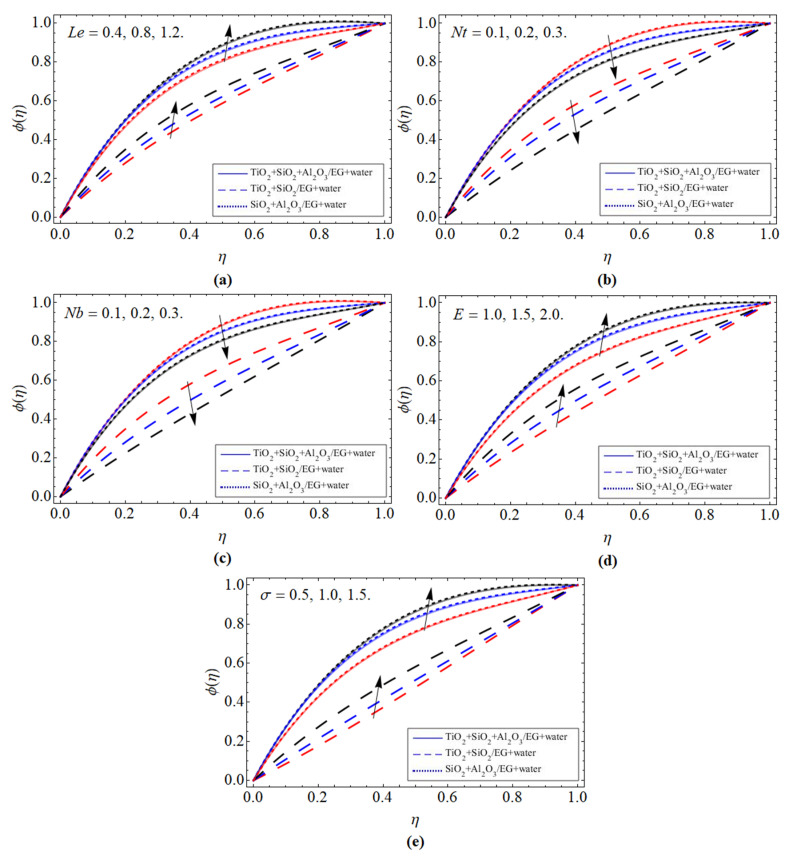
The tendency of mass profile *ϕ*(*η*) versus (**a**) Lewis number *Le*, (**b**) thermophoresis term *Nt*, (**c**) Brownian motion *Nb*, (**d**) activation energy *E*, and (**e**) chemical reaction rate *σ*.

Mass Profile *ϕ*(*η*):

Figure 5a–e displays the trend of mass profile *ϕ*(*η*) versus *Le*, *Nt*, *Nb*, activation energy *E*, and chemical reaction rate *σ*, respectively. Figure 5a–c elaborates that the transfer rate boosts with the rising values of Lewis number, which remarkably declines with the upshot of *Nt* and *Nb*. Physically, the molecular diffusion rate reduces with the variation of *Le*, which results in the reduction in the concentration boundary layer, as seen in Figure 5a. Furthermore, we are interested in investigating the influence of *Nb* and *Nt* on the flow mechanism, as these are two important factors that govern nanofluid movement. Brownian motion is a haphazard motion occurring as a result of nanomaterials in a fluid flow. Brownian motion is more powerful in fluids with low viscosity and elevated heat, as well as in fluids with tiny particles. However, their effect reduces the mass proportion ratio, as manifested in Figure 5b,c. The increment in activation energy constraint *E* and chemical reaction term dramatically elevate the mass transmission ratio, as publicized in Figure 5d,e. The effect of both factors accelerates the kinetic energy inside the fluid, which encourages fluid particles to move fast; as a result, the concentration profile of ternary nanofluid enhances.

Table 1 and Table 2 expose the experimental values of the base fluid (ethylene glycol/water) and ternary nanoparticles (TiO_2_, SiO_2_, Al_2_O_3_), and the physical model of ternary hybrid nanofluid, respectively. Table 3 reveals the statistical assessment of current results with the available work for validity purposes. It is perceived that both results show the best settlement. Furthermore, the influence of magnetic term and suction constraints enhances the skin friction of both the upper and lower plate. Table 4 exhibits the comparative valuation of present outcomes with the published literature for the Nusselt number −(*θ*′(1)).

## 5. Conclusions

We studied the properties of transient, electroviscous, ternary hybrid nanofluid flow through squeezing parallel infinite plates. The ternary HNF was manufactured by adding the TiO_2_, SiO_2,_ and Al_2_O_3_ to the conventional fluid glycol/water. The ternary hybrid nanofluid flow was modeled in the form of the system of partial differential equations, which were subsequently simplified to a set of ODEs through resemblance substitution. The obtained nonlinear set of dimensionless ODEs is further solved via the parametric continuation method. The key findings are:

- The velocity field *f*′(*η*) is reduced with the effect of the suction parameter, Darcy–Forchheimer, and magnetic field.
- The flourishing values of injection and volume friction of ternary nanoparticles (TiO_2_, SiO_2_, Al_2_O_3_) accelerate the velocity distribution.
- The electric field (*g*(*η*), *h*(*η*)) declines with the upshot of Schmidt number *Sc*, while enhancing with the increment of squeezing term *Sq*.
- The thermal energy field *θ*(*η*) is elevated versus the variation of heat source and the inclusion of nanomaterials to the base fluid, while reducing with injection effect.
- The mass allocation rate boosts with the rising values of Lewis number, activation energy constraint *E*, and chemical reaction, while declines with the upshot of thermophoresis and Brownian motion.

## Figures and Tables

**Table 1:** The experimental values of silicon dioxide $\left(\phi_{1}=\phi_{SiO_{2}}\right)$, titanium dioxide $\left(\phi_{2}=\phi_{TiO_{2}}\right)$ and aluminum oxide $\left(\phi_{3}=\phi_{Al_{2}O_{3}}\right)$ [58].

Base Fluid & Nanoparticles	*ρ* (kg/m^3^)	*k* (W/mK))	*Cp* (j/kg K)	*σ* (S/m)
C_2_H_6_O_2_-H_2_0	1063.8	0.387	3630	0.00509
TiO_2_	4250	8.953	686.2	2.38 × 10^6^
SiO_2_	2270	1.4013	3630	3.5 × 10^6^
Al_2_O_3_	6310	32.9	773	5.96 × 10^7^

**Table 2:** The physical model for ternary hybrid nanofluid [23].

Viscosity	$\frac{\mu_{Thnf}}{\mu_{f}}=\frac{1}{{\left(1-\phi_{SiO_{2}}\right)}^{2.5}{\left(1-\phi_{TiO_{2}}\right)}^{2.5}{\left(1-\phi_{Al_{2}O_{3}}\right)}^{2.5}},$
Density	$\frac{\rho_{Thnf}}{\rho_{f}}=\left(1-\phi_{TiO_{2}}\right)\left[\left(1-\phi_{TiO_{2}}\right)\left\{\left(1-\phi_{Al_{2}O_{3}}\right)+\phi_{Al_{2}O_{3}}\frac{\rho_{Al_{2}O_{3}}}{\rho_{f}}\right\}+\phi_{TiO_{2}}\frac{\rho_{TiO_{2}}}{\rho_{f}}\right]+\phi_{SiO_{2}}\frac{\rho_{SiO_{2}}}{\rho_{f}},$
Specific heat	$\frac{{\left(\rho cp\right)}_{Thnf}}{{\left(\rho cp\right)}_{f}}=\phi_{SiO_{2}}\frac{{\left(\rho cp\right)}_{SiO_{2}}}{{\left(\rho cp\right)}_{f}}+\left(1-\phi_{SiO_{2}}\right)\left[\begin{array}{l} \left(1-\phi_{TiO_{2}}\right)\left\{\left(1-\phi_{Al_{2}O_{3}}\right)+\phi_{Al_{2}O_{3}}\frac{{\left(\rho cp\right)}_{Al_{2}O_{3}}}{{\left(\rho cp\right)}_{f}}\right\}\\ +\phi_{TiO_{2}}\frac{{\left(\rho cp\right)}_{TiO_{2}}}{{\left(\rho cp\right)}_{f}} \end{array}\right]\}$
Thermal conduction	$\begin{array}{l} \frac{k_{Thnf}}{k_{hnf}}=\left(\frac{k_{Al_{2}O_{3}}+2k_{hnf}-2\phi_{Al_{2}O_{3}}\left(k_{hnf}-k_{Al_{2}O_{3}}\right)}{k_{Al_{2}O_{3}}+2k_{hnf}+\phi_{Al_{2}O_{3}}\left(k_{hnf}-k_{Al_{2}O_{3}}\right)}\right),\frac{k_{hnf}}{k_{nf}}=\left(\frac{k_{TiO_{2}}+2k_{nf}-2\phi_{TiO_{2}}\left(k_{nf}-k_{TiO_{2}}\right)}{k_{TiO_{2}}+2k_{nf}+\phi_{TiO_{2}}\left(k_{nf}-k_{TiO_{2}}\right)}\right),\\ \frac{k_{nf}}{k_{f}}=\left(\frac{k_{SiO_{2}}+2k_{f}-2\phi_{SiO_{2}}\left(k_{f}-k_{SiO_{2}}\right)}{k_{SiO_{2}}+2k_{f}+\phi_{SiO_{2}}\left(k_{f}-k_{SiO_{2}}\right)}\right), \end{array}\}$
Electrical conductivity	$\begin{array}{l} \frac{\sigma_{Thnf}}{\sigma_{hnf}}=\left[1+\frac{3\left(\frac{\sigma_{Al_{2}O_{3}}}{\sigma_{hnf}}-1\right)\phi_{Al_{2}O_{3}}}{\left(\frac{\sigma_{Al_{2}O_{3}}}{\sigma_{hnf}}+2\right)-\left(\frac{\sigma_{Al_{2}O_{3}}}{\sigma_{hnf}}-1\right)\phi_{Al_{2}O_{3}}}\right],\;\frac{\sigma_{hnf}}{\sigma_{nf}}=\left[1+\frac{3\left(\frac{\sigma_{TiO_{2}}}{\sigma_{nf}}-1\right)\phi_{TiO_{2}}}{\left(\frac{\sigma_{TiO_{2}}}{\sigma_{nf}}+2\right)-\left(\frac{\sigma_{TiO_{2}}}{\sigma_{nf}}-1\right)\phi_{TiO_{2}}}\right],\\ \;\;\;\frac{\sigma_{nf}}{\sigma_{f}}=\left[1+\frac{3\left(\frac{\sigma_{SiO_{2}}}{\sigma_{f}}-1\right)\phi_{SiO_{2}}}{\left(\frac{\sigma_{SiO_{2}}}{\sigma_{f}}+2\right)-\left(\frac{\sigma_{SiO_{2}}}{\sigma_{f}}-1\right)\phi_{SiO_{2}}}\right] \end{array}\}$

**Table 3:** The comparative assessment of present outcomes with the published literature for upper and lower plate skin friction (*f*″(1), *f*″(0)).

Parameters		*f*″(1)		*f*″(0)	
*M*	*S*	Khashi’ie et al. [47]	Present Work	Khashi’ie et al. [47]	Present Work
0.0	0.5	4.7132028	4.7132043	−7.4101525	−7.4101542
1.0		4.7391165	4.7391176	−7.5906177	−7.5906188
4.0		4.8201511	4.8201533	−8.1113342	−8.1113363
9.0		4.9647698	4.9647787	−8.9110956	−8.9110978
	0.0	1.8423469	1.8423476	−4.5868911	−4.5868933
	0.3	3.6535948	3.6535969	−6.6646620	−6.6646632
	0.6	5.3911475	5.3911494	−8.8524442	−8.8524453
	1.0	7.5933262	7.5933283	−11.9475843	−11.9475941

**Table 4:** The comparative assessment of present outcomes with the published literature for Nusselt number –(*θ*′(1)).

Parameter	Khan et al. [59] (Numerical)	Present Work	Khan et al. [59] (Analytic)	Present Work
*Sq*	$-\left(\theta^{\prime }\left(1\right)\right)$	$-\left(\theta^{\prime }\left(1\right)\right)$	$-\left(\theta^{\prime }\left(1\right)\right)$	$-\left(\theta^{\prime }\left(1\right)\right)$
0.0	−0.8443	−0.8452	−0.8438	−0.8445
0.2	−0.8791	−0.8880	−0.8783	−0.8794
0.4	−0.9151	−0.9162	−0.9140	−0.9153
0.6	−0.9523	−0.9542	−0.9511	−0.9532
0.8	−0.9908	−0.9916	−0.9914	−0.9935
1.0	−1.0306	−1.0317	−1.0310	−1.0332

## Data Availability

The data that supports the findings of this study are available within the article.
